# Illusory Recovery: Are Recovered Children With Early Language Delay at Continuing Elevated Risk?

**DOI:** 10.1044/2014_AJSLP-13-0116

**Published:** 2014-08

**Authors:** Philip S. Dale, Andrew J. McMillan, Marianna E. Hayiou-Thomas, Robert Plomin

**Affiliations:** a University of New Mexico, Albuquerque; b King’s College London, United Kingdom; c University of York, United Kingdom

**Keywords:** language disorders, development, outcomes

## Abstract

**Purpose:**

To examine the later development of language and literacy of children who had delayed language at age 2 but were in the normal range at age 4.

**Method:**

Longitudinal data were analyzed from 3,598 pairs of twins participating in the Twins Early Development Study (TEDS). Six hundred thirty-three twins (8.8%) were delayed at age 2 based on parent-reported expressive vocabulary, and of these, 373 (59.0%) were classified as recovered based on 4-year measures. Each recovered 4-year-old was matched on vocabulary, gender, and zygosity to another 4-year-old without a history of early delay.

**Results:**

Although the recovered group was below the mean for the total TEDS sample on measures of language at ages 7 and 12, there were no significant differences between the recovered and matched groups. Within the recovered group, it was not possible to predict outcome at better than a chance level.

**Conclusions:**

Children who appear to have recovered by age 4 from early delay are at modest risk for continuing difficulties, but this appears to be no higher than the risk for other 4-year-olds with equivalent scores, reflecting the continuing variability in longitudinal outcome after age 4. All children in the low normal range at age 4 merit continuing monitoring.

One of the hallmarks of early language development is variability in rate of development both between children and within a given child over time (Bates, Dale, & Thal, 1995). The former source of variability has been more intensively studied, including twin studies that estimate the relative role of genetic and environmental factors (Dale, Harlaar, Hayiou-Thomas, & Plomin, 2010; Hayiou-Thomas et al., 2006; Spinath, Price, Dale, & Plomin, 2004), as well as studies of specific aspects of the verbal environment that may play a role, including both naturally occurring parent–child interaction (e.g., Hart & Risley, 1995; Hoff & Naigles, 2002; Rowe, 2012) and clinical experiences (e.g. Girolametto, Pearce, & Weitzman, 1996; Law, Garrett, Nye, & Dennis, 2013; McCauley & Fey, 2006). Research on variability in rate over time for individual children is still largely at the descriptive stage, with some theoretical proposals such as the role of the “naming insight” (McShane, 1980) or the realization that words label categories rather than specific objects (Nazzi & Bertoncini, 2003) in driving a vocabulary spurt late in the second year of life, and the need for a “critical mass” of vocabulary for the emergence of morphosyntax somewhat later (Marchman & Thal, 2005).

A particularly intriguing phenomenon that includes both intra- and interindividual variability is early delay of language onset, often called *late talking* (Rescorla & Dale, 2013). A large and diverse body of evidence has confirmed that children with early delay are at risk for later language and literacy delays at least until adolescence, if not later (for reviews of this literature, see Hayiou-Thomas Dale, & Plomin, in press; Leonard, 1998; Rescorla & Dale, 2013; Scarborough & Dobrich, 1990). However, several longitudinal studies have found that a substantial proportion, perhaps half, of children whose language at 24–30 months is significantly delayed will have caught up by 4 or 5 years of age (Dale & Hayiou-Thomas, 2013; Dollaghan, 2013; Rescorla, 2013; Thal, Marchman, & Tomblin, 2013). This variability has both theoretical and applied significance, as it might illuminate the influences on early rate of development, as well as identify those children who would benefit from early intervention services.

Dale, Price, Bishop, and Plomin (2003) and Bishop, Price, Dale, and Plomin (2003) utilized a twin design to examine the etiology of *recovery*, that is, the predictors and causal influences on persistent versus transient delay. As the major area of language growth at 24 months is vocabulary, children with 2-year vocabulary scores below the 10th percentile based on a parent-report checklist were designated as having early language delay (ELD). At age 4, children were classified as language impaired if their scores fell below the 15th percentile on at least two of three parent-provided measures: vocabulary, grammar (see Appendix A), and a measure that included relatively abstract aspects of language use (called *abstract language* in Dale et al., 2003, but simply *language use* here; see Appendix B). Delay at 2 was a significant risk factor for delay at 4 (40.2% vs. 8.5% of the children with vs. without early delay were delayed at 4), and it can be seen that the majority of the children with early delay were not in the impaired group at 4. Although several factors, such as gender, maternal education, and nonverbal ability, were correlated with outcome, even when combined they were not able to classify individual children well enough to be clinically useful, a conclusion also drawn by Dollaghan (2013). Bishop et al. (2003) provided some evidence that persistent delay has a significantly greater genetic component than transient delay.

More recently, Hayiou-Thomas et al. (2013) showed a continuing variability in outcome from age 4 forward. Only 24.5% of the children who were delayed at age 4 (without regard to previous history) were classified as impaired on the basis of mean performance at least 1.25 *SD* (approximately the lowest 10%) on a set of four web-administered receptive language tests. The set included vocabulary, grammar, nonliteral semantics, and inferences measures. Together with the results of Dale et al. (2003), these results suggest a need to look at more complete information on early developmental measures as a potential predictor of later outcome.

In a classic article, Scarborough and Dobrich (1990) suggested that the resolution to the apparent discrepancy between recovery from early delay and continuing risk lay in their concept of *illusory recovery*. In their longitudinal study, they found that the four children with ELD had moved into the normal range by age 5, but were still at the low end of normal and had some residual weakness in selected aspects of oral language. They then went on to show severe reading difficulties at grade 2. They proposed that both early and later delays, even when they occurred in different aspects of language, were the consequence of limited language learning resources generally, not deficits in specific aspects. For this reason, each new aspect of language is mastered later and more slowly than in typically developing children. Although success will eventually occur, the underlying impairment continues and is likely to be manifested in a delay in the next stage of development. In the frequently reprinted Figure 1 of their article, there is an alternation of steep advance and plateau for both groups, but the developmental pattern is delayed—shifted to the right—for children with ELD. As a consequence, there is an alternation between similar performance in the two groups during plateaus and a divergence between the groups when typically developing children advance but the children with early delay are still at plateau. The clinical implication of this interpretation is that moving into the normal range by age 5 does not eliminate concern, both because the performance is still low, and because the early history of language delay adds to risk for later problems.

Scarborough and Dobrich’s (1990) interpretation of early delay and its apparent but often illusory recovery has been widely adopted by both researchers (e.g., Leonard, 1998) and clinicians, as it fits clinical and anecdotal evidence. Nevertheless, there has been very little empirical research on the phenomenon of illusory recovery. We do not have quantitative estimates of its frequency, that is, estimates of how many children who have apparently recovered will later have significant delays, and in which domains. And within the recovered group, we do not know if there are reliable predictors of which children are most likely to experience later difficulties. Those are the questions that motivated the present study.

A longitudinal study by Bishop and colleagues (Bishop & Adams, 1990; Bishop & Edmundson, 1987; Stothard, Snowling, Bishop, Chipchase, & Kaplan, 1998) has come closest to addressing these questions. A group of sixty-eight 4-year-olds with clinically diagnosed specific language impairment was identified (of the 87 children in the initial sample, 19 had low overall IQ scores) and the children were tested again at ages 5;6 (years;months), 8;6, and 15–16. At 5;6, the group was divided into two approximately equal groups, those who had resolved (44%) and those who had continuing poor outcome (56%). At 8;6, the resolved group continued to do well on both language and reading measures. A control group matched for socioeconomic status (SES) and gender was identified at ages 15–16. The group with continuing poor outcome at 8;6 performed significantly worse than the controls on every language measure at the later age. The resolved group continued to perform well on many measures but performed significantly poorer on nonword repetition, spoonerisms (Paddington bear => “baddington pear”) as a measure of phonological awareness, and sentence repetition. On the basis of the battery as a whole, 35% of the resolved group was judged to have impaired speech and/or language at 15–16. Group differences were even more striking on the age 15–16 reading measures: 52% of the resolved group and 93% of the continuing poor outcome group scored below the age 12 level on the reading test. Stothard et al. (1998) concluded that

if the child’s language difficulties are largely resolved by 5;6, then the outlook for spoken language development is better … however, their literacy skills were weak in relation to their peer group, possibly as a consequence of residual phonological processing impairments, including problems of phonological awareness. (p. 417)

Although the conclusions of Stothard et al. (1998) are highly consistent with research on the relation of oral language to literacy, their generalizability is limited due to three aspects of their design. First, the initial identification of language impairment was made at age 4, and the classification as resolved versus continuing at 5;6. Late talking is usually identified between 24 and 36 months, and recovery may occur by age 4. Thus, many children with resolved early delay would not have been identified in this study. Second, the study was initiated on the basis of a clinical sample; information on population-representative samples is needed to estimate the prevalence of illusory recovery. Third, only a limited analysis of predictors of later delay could be conducted given the sample size; there was some evidence that a narrative test (the Bus Story) was the best predictor of poor outcome within the resolved group. In the present study, we attempted to address these limitations. In addition, we specifically asked not only about the frequency of later language and literacy problems in children with a history of recovered ELD, but also how that frequency compared with that of a matched group of children who had not experienced early delay. That comparison is essential, because relative level of language performance can be expected to vary over time for nearly all children, for reasons of both measurement unreliability and authentic variation in rate of development.

It should be noted that because the present study, although not genetic itself, was conducted in the context of a twin design that required very large samples, there were significant constraints on language measures stemming from the impracticality of in-person testing of thousands of pairs of twins. In the preschool-age children, we used parent report measures of language that largely focused on production. At age 7, testing was conducted by telephone, which again largely focused on production, but more specifically on oral definition and on description of similarities. At age 12, we were able to use web-based testing, which was necessarily focused on receptive language.

## Design and Goals of the Present Study

For this study, we drew on the large, population-representative sample of twins with extensive longitudinal data in the Twins Early Development Study (TEDS; Oliver & Plomin, 2007). We identified a group of children (recovered) who were classified as having ELD at 2 years but were well out of the clinical range at age 4. We compared them with a group of 4-year-olds who were matched on the basis of gender, zygosity, and age 4 vocabulary, but who had not shown ELD at age 2. The matching was needed because the recovered group was still below the population mean on most measures; this is a typical finding for children with ELD (see Rescorla & Dale, 2013, for a summary of that research). We examined both word decoding and reading comprehension skills at ages 7 and 12 in addition to later language development at these ages, because literacy skills have been consistently found to be lower in children with a history of language impairment than for children without such a history (Bishop & Snowling, 2004; Kamhi & Catts, 2011). On both theoretical and empirical grounds, however, we expected reading comprehension to be more likely to be affected than word decoding by a history of language difficulties (Harlaar et al, 2010; Hoover & Gough, 1990). We addressed the following questions:

1. How do children with ELD at age 2 who are back in the normal range at age 4 perform relative to the whole sample with respect to (a) language development at age 7, (b) word decoding at age 7, (c) receptive language at age 12, (d) word decoding at age 12, and (e) reading comprehension at age 12?
2. Do the outcomes for the recovered group differ from those for a matched group of 4-year-olds with no history of ELD with respect to those same five measures, in terms of both mean level and the proportion of children in the clinical range (−1.25 *SD*)?
3. Is it possible to predict who is most likely to have these problems, that is, performance in the clinical range, using gender, family history of language/literacy difficulties, maternal education, severity of ELD, and level of vocabulary, grammar, and language use at age 4?

## Method

### Participants

The broad sampling frame for the present study was TEDS, a longitudinal study of twins born in England and Wales in 1994, 1995, and 1996 (Haworth, Davis, & Plomin, 2013; Oliver & Plomin, 2007). The twins were assessed at 2, 3, and 4 years of age using parent questionnaires that included measures of language, cognitive, and behavioral development. They have continued to be assessed in these and other domains with a variety of methods including telephone assessment; parent-administered tests; teacher National Curriculum ratings; and increasingly from age 10 on, web-based assessment. Twin pairs were excluded if either member had a genetic disorder such as Down syndrome, any major medical or perinatal problems, documented severe hearing loss or blindness, organic brain damage, or a neurodevelopmental disorder such as autism spectrum disorder. Only participants for whom English was the first language spoken at home were selected. A total of 3,598 pairs (1,233 monozygotic; 1,196 dizygotic same sex; 1169 dizygotic opposite sex) met the inclusion criteria listed above, and had complete language measures at ages 2 and 4 years, essential for defining the set of children with ELD, and further dividing that set into those with recovery versus those with continuing difficulties.

The TEDS sample has continued to be reasonably representative of the UK population with respect to ethnicity, maternal education and employment, and paternal employment (see Haworth et al., 2013, for an overview of sample representativeness), although by adolescence the sample has somewhat higher maternal education and a higher proportion of white families than at study entry. In the present sample, which was selected to be ethnically white in order to enable later molecular genetic analysis, the proportion of mothers with at least A-level qualifications (age 18 exam, generally required for university entrance) was 38.9%, compared with 32%, respectively, in the UK population (Walker, Maher, Coulthard, Goddard, & Thomas, 2001).

All phases of this project were reviewed and approved by the Institute of Psychiatry Ethics Review Board. Parental consent was obtained before data collection.

### Measures

#### Language at age 2 and the definition of ELD

Parents completed the MacArthur Communicative Development Inventory: U.K. Short Form (MCDI:UKSF; Dionne, Dale, Boivin, & Plomin, 2003). This instrument includes a list of 100 words from which parents are asked to check those that they have heard their child say. The 100 words were selected (Fenson et al., 2000) from the 680 words on the longer MCDI (Fenson et al., 1994) to give good prediction of the total score on the latter. The list of words was then Anglicized for appropriate spelling in a UK setting. Following Dale et al. (2003), a cutoff of 15 or less was used to define ELD. Of the 7,196 individual children, 633 (8.8%) met this criterion for ELD. (This is somewhat lower than the 9.6% reported in Dale et al. [2003] due to the fact that some children with low early scores were eliminated from the present sample as a result of later diagnosis of autism and other neurodevelopmental disorders.) In 440 of these cases, both the twin and co-twin were classified as delayed; that is, the children with early delay included both twins in 220 pairs and 193 twins whose co-twin was not delayed.

#### Language at age 4 and the definition of recovered and nonrecovered delay

Again following Dale et al. (2003), multiple aspects of language were assessed at age 4. They included a vocabulary checklist of 48 words chosen on the basis of literature review and pilot testing, a question about sentence development with six levels of response (see Appendix A), and a set of 14 questions concerning the child’s receptive and expressive mastery of more abstract concepts (see Appendix B). For each of these three dimensions, a cutoff was determined that approximated the lowest 15% of children: less than 29 for Vocabulary, less than 6 for Grammar, and less than 8 for Abstract Language. Rather than averaging the three dimensions, an overall criterion for delay at 4 years was the presence of delay in at least two of the three. This decision rule was established to ensure sensitivity to impairment in a child with an uneven language profile. For the present study, it was desirable to have a stricter criterion for recovery than absence of delay as defined above. Recovery was defined as the absence of delay in any of three dimensions. Of the 633 children classified as having ELD, 373 (59.0%) met the criterion for recovery. In 218 of these cases, both the twin and co-twin were classified as recovered; that is, there were 109 twin pairs in whom this occurred. Table 1 summarizes information about the two groups of interest for this study, as well as the remaining group of children with ELD who had not recovered at age 4.

#### Language at age 7

Two verbal measures were administered by telephone. They were the Vocabulary (what does “strenuous” mean?) and Similarities (in what way are milk and water alike?) subtests of the Wechsler Intelligence Scale for Children—Third Edition (WISC–III–UK; Wechsler, 1992). (See Kovas, Haworth, Dale, and Plomin, 2007, for more information about telephone administration.)

#### Word decoding at ages 7 and 12

Twins were assessed separately on the Test of Word Reading Efficiency (TOWRE; Torgesen, Wagner, & Rashotte, 1999) by telephone at ages 7 and 12. The TOWRE has two timed (45-s) subtests, Phonological Decoding Efficiency (PDE), which requires reading decodable nonwords (pseudowords such as *tegwop*), and Sight Word Reading (SWE), which requires reading real words. Word and nonword stimuli were mailed to twins in advance of the telephone testing session with instructions that they should not be opened prior to the test session. The SWE and PDE subtests are strongly correlated (0.83 at age 7, 0.74 at age 12). For this reason, all subsequent analyses were conducted on overall TOWRE scores, calculated as the mean of PDE and SWE scores at each age.

#### Receptive language at age 12

At 12, participants were assessed on a web-based set of four language measures (Dale et al., 2010), all of which are subtests of well-established published test batteries, whose manuals report details of test validation and reliability. The development of the web-based battery, as well as details of the testing procedures, are reported in Kovas et al. (2007).

#### Vocabulary

The WISC–III–PI Vocabulary Multiple Choice subtest (Wechsler, 1992), was administered at age 7.

#### Nonliteral semantics

In addition to vocabulary, semantics was assessed using the Figurative Language subtest of the Test of Language Competence—Expanded Edition, Level 2 (Wiig, Secord, & Sabers, 1989). This subtest assesses the interpretation of idioms and metaphors; correct understanding of such nonliteral language requires rich semantic representation as well as an awareness of the ambiguity of many expressions between their literal and figurative meaning. The child heard a sentence orally and chose one of four answers, presented in both written and oral forms.

#### Syntax

Syntax was assessed using the Listening Grammar subtest of the Test of Adolescent and Adult Language—Third Edition (Hammill, Brown, Larsen, & Wiederholt, 1994). Children were required to select two sentences that have nearly the same meaning from a set of three options. The sentences were presented auditorily only.

#### Pragmatics

The Making Inferences subtest of the Test of Language Competence—Expanded Edition, Level 2 (Wiig et al., 1989) requires participants to make permissible inferences on the basis of existing, but incomplete, causal relationships in the context of short paragraphs presented orally. The child chose two of four responses, presented in both written and oral form, that best explained what could have happened.

#### Reading comprehension at age 12

Twins completed two measures of reading comprehension: the Reading Comprehension subtest of the Peabody Individual Achievement Test (PIAT_rc_; Markwardt, 1997) and the GOAL Formative Assessment in Literacy for Key Stage 3 (Global Online Assessment for Learning, 2002). The PIAT_rc_ assesses literal comprehension of sentences. Children were required to read each sentence and were then shown four pictures. They had to select the picture that best matched the sentence they had read. The GOAL assesses both literal and inferential reading comprehension. Questions are grouped into three categories: Assessing Knowledge and Understanding (e.g., identifying information, use of punctuation and syntax), Comprehension (e.g., grasping meaning, predicting consequences), and Evaluation and Analysis (e.g., comparing and discriminating between ideas). Within each category, questions about words, sentences, and short paragraphs are asked. Because we were primarily interested in comprehension skills, we used questions from the two most relevant categories, Comprehension, and Evaluation and Analysis, with 20 items from each category. Correct answers were summed to give a total comprehension score. For both the PIAT_rc_ and the GOAL web-based tests, an adaptive algorithm modified item order and test discontinuation depending on the performance of the participant. Both tests contained the same practice items, test items, and instructions as the original published tests.

For all five of the ages 7 and 12 outcome measures, low performance was defined as a score at least −1.25 *SD* below the mean of the TEDS sample.

#### SES and family history of language/reading difficulties

A composite measure of SES, used in many TEDS publications (Pike, Iervolino, Eley, Price, & Plomin, 2006), was based on a combination of occupational status and education for both parents. In addition, based on a questionnaire completed by parents when their twins were age 9, we identified families in which a first-degree relative (father, mother, brother, sister) was reported to have had difficulties in learning to speak, or to read.

### Analysis

In order to conduct the most appropriate comparison of children with recovered ELD with children who had not experienced ELD (Research Question 2), a matching design was used. Each 4-year-old in the recovered group was matched with another 4-year-old with respect to gender, zygosity, and age-4 vocabulary, and paired-sample *t* tests were used to compare the two groups. For the third research question, which concerned the prediction within the recovered group of which children would experience further difficulties, we used logistic regression for each of the five outcome variables. For each analysis, the independent variables included gender; family history of language/literacy difficulties; maternal education; severity of early delay; and level of vocabulary, grammar, and language use at age 4.

Following the identification of the recovered and matched groups, all later analyses were conducted on the basis of one randomly selected twin from each pair, in order to preserve independence of data. This is commonly done in phenotypic (nongenetic) analyses of twin data. However, due to the centrality of the comparison of the recovered and matched groups on later measures for our overall research goal, we also conducted a mixed model analysis for those comparisons that took account of the nesting of twins within families. This maximized the power of the tests by using all the data. As will be shown below, this did not change the pattern of results.

## Results

Table 1 presents a comparison of the recovered ELD group with the matched comparison group with respect to the age 4 measures as well as gender, zygosity, SES, and family history of language/literacy difficulties. As intended by the design of the study, the two groups differed significantly on age 2 vocabulary, but did not differ significantly on age 4 grammar and language use. Descriptive information is also provided for the group with ELD who did not recover by age 4. As expected, they scored consistently lower on all measures at 2 and 4 years, were somewhat more likely to be male, and have lower SES ratings. Table 2 summarizes comparisons of the recovered and the matched comparison groups on the five dependent variables of the study, including comparisons of mean level, and the proportion of children with later difficulties in the clinical range (defined above as at least 1.25 *SD* below the mean). These are *z* scores, for which 0 represents the mean, and 1 the *SD*, of the full TEDS sample, which as noted earlier is reasonably representative of the U.K. population.

### Question 1: Comparison of Recovered Sample With TEDS Sample as a Whole

As can be seen in Table 2, the recovered sample continued to score below the TEDS mean on oral language at age 7 and receptive language at age 12 (see negative *z*-scores), but the effect size was small, only about 0.05 *SD*. For all three reading measures, in contrast, the recovered group scored slightly higher than the TEDS mean. Overall, these results, especially the *SD*s, which are equivalent to that of the entire TEDS sample, document the great variability in outcome of the recovered group.

### Question 2: Comparison of Recovered Sample With Matched Sample

**Figure 1:**
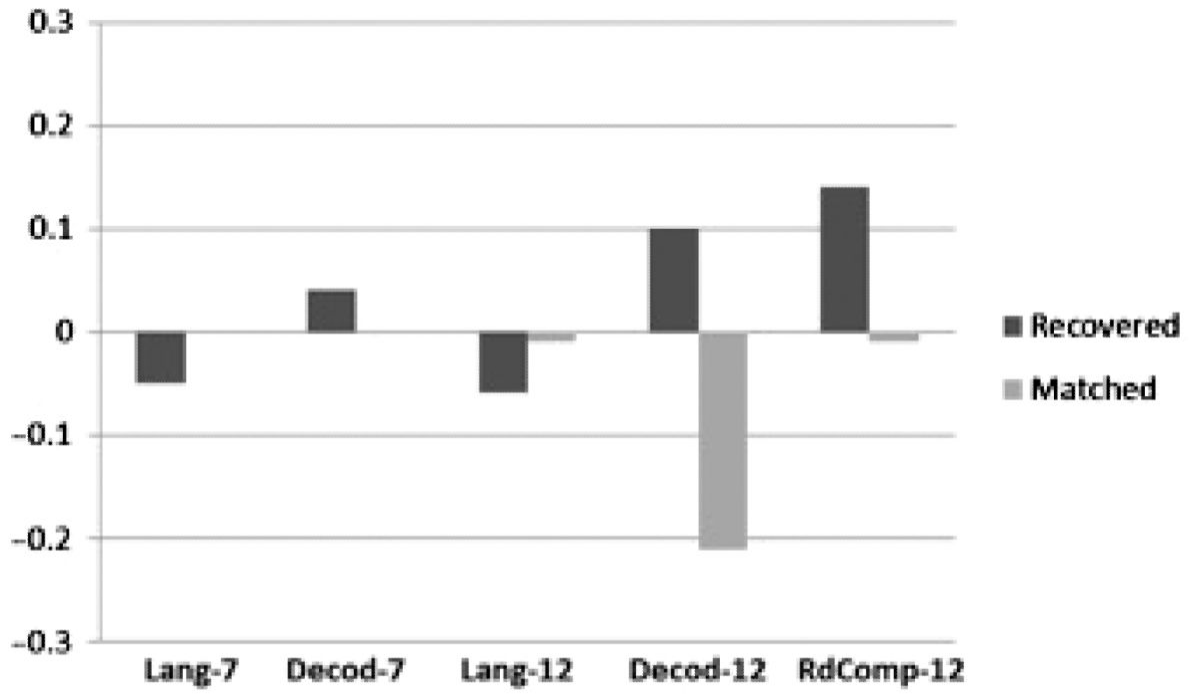
Comparison of recovered and matched groups on mean standardized language and reading outcome measures at ages 7 and 12. Lang-7 = language at age 7; Decod-7 = word decoding at age 7; Lang-12 = language at age 12; Decod-12 = word decoding at age 12; RdComp-12 = reading comprehension at age 12.

As also shown in Table 2, the recovered group scored lower than the matched group on the measure of oral language at age 7 and on receptive language at age 12. However, in neither case was the difference significant, and the effect sizes were very small. For the three reading measures, the recovered group scored higher than the matched group, but in none of the cases was this significant. Figure 1 presents these results graphically. To further explore the possibility of differences between the recovered and matched groups, we used mixed model analyses in SPSS to compare outcome measures for the two groups. The analysis, analogous to a paired-sample *t* test, is based on the difference between each pair of matched twins, and tests the null hypothesis that the mean such difference is equal to zero. For these analyses, all twins in the two groups could be used, as the analysis allows for the nesting of twins within families. Because the larger set of twins was used, there is additional statistical power. However, as shown in Table 3, there are still no significant differences between the groups on any of the later measures.

In addition to the comparison of mean scores for the two groups, we examined the proportion of children with low scores (< −1.25 *SD*). For none of the five outcome measures was this proportion of children significantly different for the recovered and matched groups (all *p*s > .3). For comparison, the proportion of the entire TEDS sample used in this article with low scores (< −1.25 *SD*) on each of the later measures in Table 2 was 10.5%, 8.3%, 9.1%, 8.6%, and 9.2%, respectively. The figures for the recovered group are quite comparable.

### Question 3: Prediction of Later Impairment

The analyses reported above focus on the recovered group as a whole. To address the third research question, our ability to identify the children most likely to show later problems, we conducted a logistic regression analysis predicting placement in the clinically delayed group for each outcome measure. (For these analyses, family history was not entered as a predictor, as a simple chi-square analysis confirmed that it was not related to any of the outcomes. In addition, for most of the outcome measures, there was a cell with a zero entry, which renders the estimate of B indeterminate.) On the whole, very few significant predictors were found in these analyses, which are summarized in Table 4. SES was a predictor of low language at ages 7 and 12, and word decoding at age 7, with higher SES resulting in lower odds of low performance on those measures (and hence odds ratios of less than 1). Sex was a significant predictor of low score on oral language at age 7. With the exception of SES as a predictor of low receptive language at age 12, the odds ratios were generally not substantial enough to have any clinical value.

## Discussion

Children with early delay whose scores are in the normal range at 4 years are still somewhat below average at that age, a finding which has been noted in other studies (Rescorla, 2013). Thus it is not surprising that, on average, they continue to score below average on measures of oral language at age 7 and receptive language at age 12, though the deficit is small, less than 0.1 *SD* (Research Question 1). However, in contrast to Scarborough and Dobrich (1990), who observed the most substantial effect of a history of ELD on reading, our children showed weaknesses in oral language, but not reading. In fact, the recovered group scored higher on all three reading measures than the TEDS sample as a whole, though the differences were very small (*d* < 0.15). The lack of impairment on word decoding at age 7 is not surprising, as decoding is less closely related to language than comprehension. Scarborough and Dobrich’s measure of reading at grade 2, the Woodcock-Johnson Psychoeducational Battery, is a composite of word reading and prose comprehension. It is less clear why we did not observe a deficit on a measure of reading comprehension at age 12, but it is likely that this reflects continuing variability in both language and reading after the age of 4, especially over an 8-year interval (Hayiou-Thomas, Dale & Plomin, 2012).

The striking finding of the present study is that when these children are matched with other 4-year-olds at the same level of vocabulary and grammar, there is little evidence for an elevated continuing risk (Research Question 2). There were no significant differences with respect to either mean score or falling in the clinical range on any of the measures, and effect sizes were very small (*d* < 0.2), with the exception of word decoding at age 12, for which the effect was small (*d* = 0.28), but in the opposite direction from that predicted. The results do not provide evidence for the phenomenon of illusory recovery, that is, an elevated risk for later difficulties relative to level of functioning at age 4. Instead, there is clear evidence for both continuing risk for low normal performance at age 4 regardless of history, and for substantial variability in outcome for children. Expressed somewhat differently, the great variability in outcome observed from 2 to 4 years is not the end of the story.

The failure to find evidence for illusory recovery in the form of differences between the recovered and matched groups was counter to expectations and merits careful evaluation. Is it possible that illusory recovery occurs but is masked by features of the present research design? One possibility is that the recovered group had stronger skills than a truly representative recovered group would have had. The use of a low extremes cutoff for a measure with imperfect reliability implies that a certain amount of regression to the mean may have occurred, and that some children in this group may not have had ability below the age 2 cutoff for ELD. Nevertheless, even if this occurred, the recovered group should have been, on average, below the matched group at age 2. Furthermore, the use of a measure at age 4 to define recovery that was sensitive only to vocabulary and grammar makes it very likely that some of this group were not entirely recovered, in that they may have had enduring difficulties in semantics, pragmatics, or other aspects of language. Thus this first possibility seems unlikely. An alternative possibility that would lead to no differences in the present analysis, even if illusory recovery occurs, is that the present matched group was less capable than a perfectly representative matched group would have been. However, the mean vocabulary at age 2 of the matched group (49.1; Table 1) was virtually identical to that for the full sample of 7,196 twins (*M* = 48.43, *SD* = 24.56, *z* = .03). Their age 4 vocabulary was, as noted earlier, somewhat lower than this. Compared with the full sample, with *M* = 37.02, *SD* = 8.16, the mean *z* for 4 year vocabulary was -.24. Taking age 2 vocabulary into account, the matched group may have been in fact slightly more capable than a perfectly matched group. We conclude that the finding of no difference is likely to be a valid one.

Stothard et al. (1998) estimated that 35% of their resolved group had speech-language impairment at ages 15–16. In contrast, we found only about 14% were in the low extreme at age 12. Comparison is difficult, however, as each study used its own measures and perhaps more importantly, its own criterion for low performance. Stothard et al. acknowledged using a stringent criterion for normality: “Children were regarded as having a good outcome only if they were essentially indistinguishable from controls” (p. 413). This meant no score more than 1.89 *SD* below the mean, and at most one score more than 1.29 *SD* below the mean. Hence a larger proportion of their children would be classified as impaired than in the present study, which utilized a criterion of −1.25 *SD*. It is also possible, however, that impairments become increasingly substantial as children move into adolescence, and therefore are genuinely larger at ages 15–16 than at age 12.

We were unable to identify those children within the recovered group who were likely to show later delays on any of the five measures at ages 7 and 12 (Research Question 3). The closest thing to a pattern was that low SES was a significant predictor of low performance on the language measures at ages 7 and 12, and word decoding at age 7. This may be due in part to the limited measures available for use as predictors at age 4 in this study, given its heavy reliance on parent report. However, this negative result for the second research question mirrors the conclusion of numerous studies of recovery from ELD. It would be exceptionally valuable for clinicians providing services to young children that apparently succeed in producing recovery if they could identify a subset of children who should receive ongoing monitoring. In the absence of such results, we believe that all children with documented ELD and apparently normal performance in the range of 4–6 years of age should be periodically screened for signs of later delay. In addition, other children in the low normal range might usefully be screened early in the elementary school years, perhaps by parent report or teacher-administered measures.

A surprising aspect of the results was the good performance of the recovered group on the TOWRE at age 12. Because we made a directional prediction of superior performance by the matched group, this difference cannot be considered statistically significant, but the effect size is notable. Several previous studies have found relatively good decoding skills in at least a subset of children with language impairment, whether the decoding measure used was based on pure accuracy (Catts, Adlof, Hogan & Weismer, 2005) or was based on fluency such as the TOWRE (Bishop, McDonald, Bird & Hayiou-Thomas, 2009). Bishop et al. found that rapid serial naming was a key predictor of good decoding, along with unimpaired phonological processing skills. Nevertheless, in those studies it was never the case that the impaired group performed better than the typically developing group. The present study is not entirely parallel to Bishop et al. (2009) though; both the recovered group and the matched comparison group were apparently unimpaired at age 4. Although this particular result remains puzzling, overall the pattern of results is consistent with previous research in demonstrating that some history of language impairment, even if apparently recovered, has a greater negative impact on reading comprehension than on word decoding.

Several limitations to the generality of our conclusions should be noted. The first is that the present data are from twins. Although twinning is known to be associated with delay in early language milestones, the results of research on twins, including the balance of persistent versus transient problems, are generally very similar to those from singletons (cf. Dale et al., 2003, for a review). Second is the absence of an early measure of language comprehension. Several studies have found significant prediction from early comprehension measures to later language development (e.g., Thal et al., 2013), although the prediction is not accurate enough to be clinically useful. However, it may be that measures of comprehension in the children with early delay would add to the identification of children in the recovered group at most risk for later problems.

Perhaps the most intriguing, but challenging, issue is the role of therapeutic intervention in the developmental trajectory of language. Does receiving therapy between ages 2 and 4 add to the prediction of long-term outcome, either positively or negatively? Two methodological problems prevent us from addressing that question. The first is that our information on therapy in the present sample is both incomplete and too coarse (in both the exact age of therapy, and the focus of the therapy) to be informative. The second is a more general problem. The children who are most likely to receive therapy are those with more serious problems; for this reason, when analyzed by itself in a regression analysis, therapy is likely to predict worse outcomes. The best currently available tool for addressing this kind of issue in nonexperimental research is *propensity score matching* (Shadish, Cook, & Campbell, 2002), This is a two-stage analysis. In the first stage, a model is built to predict which children will receive therapy. It is essential to be able to do this with relative accuracy before moving to the next stage, in which each child who receives therapy is matched to another child with the same composite propensity score who did not receive therapy. If the propensity scores do not have high validity (i.e., predict therapy accurately), the difference between the two groups is meaningless. We do not have at present such a model of therapy involvement, and it is likely that a successful model will require a rich and detailed body of information about both child and family. Consequently, we cannot at present know whether the recovery that occurred for a large proportion of the twins would have occurred spontaneously or was the result of therapeutic intervention (but see Finestack & Fey, 2013, for suggestions on clinical management of late talkers).

If, as the present results suggest, illusory recovery in the sense of a continuing elevated risk for children whose performance has moved to the normal range does not actually occur, we may ask why it is such a widely accepted construct. We suggest that it is an example of a deeply rooted and frequently active aspect of human cognition: the search for patterns, including patterns of association between discrete events. An extensive body of research (see Fiedler, 2004) has documented the tendency of observers to see correlations that are not there. This phenomenon has been called *illusory correlation*. Sometimes these erroneous conclusions are driven by expectancies or stereotypes, as in the tendency to recognize and give added weight to instances of particular behaviors being associated with specific ethnic groups or genders. In other cases, they result from unequal weighting of information, in particular, the tendency to pay greater attention to cases when both a presumed cause and a presumed effect are both present than the other three cases (this can also be seen as an example of the well-documented confirmation bias). We suspect that both of these processes may have occurred in clinical thinking, but especially the latter. When a child is seen in the school years with a language difficulty, and the clinician has treated the child earlier with good results, or the case history tells a similar story, this is a positive example of the connection and is likely to stay in the clinician’s mind. An awareness, perhaps even expectancy, of the concept of illusory recovery may facilitate this bias further. Cases of children who are treated successfully and never seen again, or those who have no early history but are seen clinically later in childhood, simply do not make the same impression on the clinician’s mind.

We hope that the present study will help clinicians recognize that the relevant category of children who merit concern and monitoring is not the recovered group, but all children in the low normal group, regardless of early history. Such an approach is consistent with the growing acceptance of a dimensional, rather than categorical, conception of language disorder (Dollaghan, 2004; Leonard, 2013; Rescorla, 2013). We have not yet identified clinically useful predictors of later problems. Low SES adds slightly to the prediction of later oral language skills and word decoding at age 7, but nothing to the prediction of reading difficulties at age 12. The search for useful predictors of later language and literacy skills should be one of the highest priorities for future research.

## Figures and Tables

**Table 1:** Comparison of recovered group with matched comparison group on demographic and early language measures.

Measure	Recovered ELD		Matched, not ELD					ELD, not recovered	
	*M (SD)*	*n*	*M (SD)*	*n*	*t*	*P*	*d*	*M (SD)*	*n*
Initial definition of groups, using all recovered twins									
Vocabulary at 2	10.3 (3.6)	373	49.1 (20.7)	373	— ^a^		3.2	8.8 (4.0)	260
Vocabulary at 4	35.0 (6.8)	373	35.0 (6.8)	373	— ^b^		0.0	20.7 (9.7)	260
Restricted sample, based on one randomly selected twin from each pair									
Vocabulary at 2	10.1 (3.7)	240	47.9 (19.6)	190	−0.26 (199.5)	< .001	3.5	9.0 (3.9)	140
Vocabulary at 4	35.5 (6.7)	240	35.2 (6.9)	190	0.53 (428)	.59	0.04	21.1 (9.5)	140
Grammar at 4	5.9 (0.36)	240	5.9 (0.32)	189	−0.26 (427)	.80	0.0	4.8 (0.93) 136	140
Language use at 4	9.8 (2.1)	230	10.0 (0.26)	177	−0.88 (334.6)	.38	0.15	4.7 (2.6)	122
% male^d^	62.1		64.2			.36		70.4	
% MZ:DZss:DZos	40.4: 28.3: 31.3		41.6: 28.4: 30.0			.65		41.9: 30.3: 27.7	
SES	0.046 (.95)	217	0.040 (0.96)	182	0.057 (397)	.95	.01	−0.26 (0.90)	127
% Family history^c,d^	3.8		7.4			.13		3.8	

*Note*. MZ = monozygotic; DZss = dizygotic, same sex; DZos = dizygotic, opposite sex; SES = socioeconomic status.

a Unequal (and nonoverlapping) by design.

b Matched by design.

c First-degree relative with history of language/literacy learning difficulties.

d Fisher’s exact test, two-sided.

**Table 2:** Comparison of recovered group with matched comparison group on five language and literacy outcomes measures.

Measure (*n* pairs)	Recovered *M (SD)*	Matched *M (SD)*	*t (df)*	*p*	*d*	Recovered (% low)	Matched (% low)	*p*
Oral language at age 7 (115)	−0.05 (0.84)	.00 (1.05)	−0.48 (114)	.63	0.05	10.4	9.6	.32
Word decoding at age 7 (114)	0.04 (0.91)	.00 (0.89)	0.29 (113)	.78	0.04	6.1	8.8	.52
Receptive language at age 12 (51)	−0.06 (1.04)	−0.01 (0.92)	−0.24 (50)	.82	0.05	13.7	11.8	.39
Word decoding at age 12 (49)	0.10 (0.90)	−0.21 (0.95)	1.83 (48)	.07	0.28	6.1	16.3	.58
Reading comprehension at age 12 (69)	0.14 (0.94)	−0.01 (1.10)	0.95 (68)	.35	0.15	7.2	14.5	.56

*Note*. Analyses in this table are based on paired-sample *t* tests, or Fisher’s exact tests, based on one randomly chosen twin per pair.

**Table 3:** Mixed model comparison of recovered group with matched comparison group on five language and literacy outcomes measures.

	Difference between recovered and matched twins *M (SD)*			Fit parameters		
Measure (*n* pairs)		*t*	*P*	−2LL	AIC	BIC
Oral language at age 7 (218)	−0.154 (1.26)	−1.51	.133	718.4	722.4	729.2
Word decoding at age 7 (214)	0.008 (1.25)	0.066	.947	700.8	704.8	711.6
Receptive language at age 12 (95)	−0.21 (1.29)	−1.81	.075	314.3	318.3	323.4
Word decoding at age 12 (93)	0.197 (1.16)	1.56	.123	292.3	296.3	301.3
Reading comprehension at age 12 (131)	−0.120 (1.37)	−1.16	.247	450.6	454.6	460.4

*Note*. LL = log likelihood; AIC = Akaike information criterion; BIC = Bayesian information criterion.

**Table 4:** Results from logistic regression analyses predicting placement in the low group on five language and literacy outcomes measures for children in the recovered group (separate univariate analyses).

Outcome Measure (*n* pairs)	Sex		SES		Voc-2		Voc-4		Gram-4		Abstr-4	
	*B (SE)*	*OR*	*B (SE)*	*OR*	*B (SE)*	*OR*	*B (SE)*	*OR*	*B (SE)*	*OR*	*B (SE)*	*OR*
Oral language at age 7^a^ (151)	1.04* (0.51)	2.8	−0.67* (0.28)	0.51	−0.09 (0.07)	0.91	−0.02 (0.03)	0.98	0.54 (1.07)	1.71	0.06 (0.13)	1.06
Word decoding at age 7^b^ (151)	−0.62 (0.77)	0.54	−0.82* (0.38)	0.44	−0.07 (0.09)	0.93	0.08 (0.05)	1.09	— ^c^		0.00 (0.18)	1.00
Receptive language at age 12^d^ (104)	1.00 (0.75)	2.71	−1.76* (.62)	0.17	−0.02 (0.10)	0.98	0.06 (0.05)	1.06	1.13 (1.31)	3.10	−0.27 (0.22)	0.76
Word decoding at age 12^e^ (102)	— ^c^		−0.87 (0.99)	0.42	−0.11 (0.17)	0.89	0.00 (0.08)	1.00	— ^c^		0.00 (0.41)	1.00
Reading comprehension at age 12^f^ (122)	0.70 (0.63)	2.0	−0.13 (0.37)	0.88	0.13 (0.10)	1.14	0.03 (0.05)	1.03	— ^c^		−0.07 (0.17)	0.94

*Note*. Analyses are based on one randomly chosen twin per pair. Voc-2 = age 2 vocabulary (severity of early delay); Voc-4 = age 4 vocabulary; Gram-4 = Grammar score at 4; Abstr-4 = abstract language use at 4; *OR* = odds ratio.

a Cox and Snell *R*^2^ = .077; Nagelkerke *R*^2^ = .139; model ×^2^(6) = 12.09,*p* = .06.

b Coxand Snell *R*^2^ = .077; Nagelkerke *R*^2^ = .188; model ×^2^(6) = 12.02, *p* = .06.

c Parameter cannot be estimated due to a low cell frequency or high multicollinearity

d Cox and Snell *R*^2^ = .156; Nagelkerke *R*^2^ = .319; model ×^2^(6) = 17.70, *p* = .007.

e Cox and Snell *R*^2^ = .043; Nagelkerke *R*^2^ = .185; model ×^2^(6) = 4.50, *p* = .61

f Cox and Snell *R*^2^ = .048; Nagelkerke *R*^2^ = .102; model ×^2^(6) = 6.06, *p* = .42.

* *p* < .05.

## Appendix A. Grammar Rating at 4 Years

**Table:**

Parents were asked to select one of the following in response to the instruction, “On the whole, which of the following best describes the way your child talks?”
1. Not yet talking
2. S/he is talking, but you can’t understand him/her
3. Talking on one-word utterances such as “milk” or “down”
4. Talking in 2 to 3 word phrases, such as “me got ball” or “give doll”
5. Talking in fairly complete sentences such as “I got a doll” or “can I go outside?”
6. Talking in long and complicated sentences, such as “when I went to the park, I went on the swings,” or “I saw a man standing on the corner”

## Appendix B. Language Use at 4 Years

**Table:**

1. Can your child say how old s/he is?
2. Can your child say the month and day of his/her birthday when asked?
3. Can your child tell you what happened at a past event (such as birthday party or holiday), as if s/he were telling a story from beginning to end?
4. Can your child talk clearly about what s/he will do later on, such as tomorrow or next week?
5. Can your child tell a fairy tale, joke, or television show story completely from beginning to end and in the correct order?
6. Does your child know his/her right hand from his/her left?
7. Does your child use -est words, like biggest, strongest, greatest?
8. Does your child use the word “today” correctly?
9. Does your child use the word “yesterday” correctly?
10. Does your child understand the difference between “accident” and doing something “on purpose”?
11. Does your child ever ask you what a word means?
12. Does your child use phrases or sentences containing “but”?
13. Does your child talk about the order of events by using words like “before” and “after”?
14. Does your child “play” with language by making jokes about words and their sounds, such as words that rhyme?
